# Effect of Treadmill Exercise and Probiotic Ingestion on Motor Coordination and Brain Activity in Adolescent Mice

**DOI:** 10.3390/healthcare9010007

**Published:** 2020-12-23

**Authors:** Junechul Kim, Bo-Eun Yoon, Yong Kyun Jeon

**Affiliations:** 1 Graduate School of Physical Education, Dankook University, Yong-in 16860, Korea; jkim1@tamusa.edu; 2 Department of Molecular Biology, Dankook University, Cheonan 31116, Korea

**Keywords:** treadmill exercise, probiotics, motor function, cytokines, GABA, glutamate

## Abstract

High-intensity exercise can lead to chronic fatigue, which reduces athletic performance. On the contrary, probiotic supplements have many health benefits, including improvement of gastrointestinal health and immunoregulation. However, the effects of probiotics combined with exercise interventions on motor functions and brain activity have not been fully explored. Therefore, this study aimed to identify the effects of probiotic supplements and aerobic exercise on motor function, immune response, and exercise intensity and probiotic ingestion. After four weeks of intervention, the motor functions were assessed by rotarod test, then the levels of cytokines, gamma-aminobutyric acid (GABA), and glutamate were detected. The improvement caused by the intake of probiotics in the moderate-intensity exercise group and the non-exercise group in the accelerating mode rotarod was significant (*p* = 0.038, *p* < 0.001, respectively). In constant-speed mode, the moderate-intensity exercise group with probiotic ingestion recorded longer runs than the corresponding non-exercise group (*p* = 0.023), and the improvement owing to probiotics was significant in all groups—non-exercise, moderate, and high-intensity (*p* = 0.036, *p* = 0.036, *p* = 0.012, respectively). The concentrations of inflammatory cytokines were lower, whereas GABA was higher in the probiotics-ingested group. Taken together, exercise and probiotics in adolescence could positively affect brain and motor function.

## 1. Introduction

Exercise for growth and development in adolescence is essential [1], and it has been reported that moderate-intensity exercise in this period is highly beneficial [2]. Adolescent boys experience various athletic strengths, ranging from moderate to high-intensity. However, high-intensity exercise can sometimes harm immunity, which increases the risk of infection [3], causing an increase in oxidative stress and active oxygen [4]. Moreover, continued exposure to excessive training also causes chronic fatigue and reduces athletic performance [5].

Recently, probiotics have been reported to improve growth, motor ability, and brain function. For example, *Veillonella* was found to be more abundant in marathoners’ intestines than in those of the general population, and research has shown that mice injected with *Veillonella atypica* exhibited increased endurance [6]. In addition, earlier studies have shown that the intake of *Lactobacillus plantarum* twk10 increased adult endurance [7], whereby the intake of *L. plantarum* ps128 during high-strength training improved the motor skills of triathlon athletes [8]. Previous studies have indicated that probiotics significantly influence immune function [9,10] by increasing immune cell proliferation and sensitivity to external antigens [11,12]. Besides this, probiotic ingestion led to changes in the neurotransmitters involved in exercise and cognitive functions [13,14], and enhanced learning and memory in treated mice [15]. Based on the reports that probiotics increase the production of gamma-aminobutyric acid (GABA), a major inhibitory neurotransmitter in the central nervous system [16], to balance brain excitation and inhibition and to improve cognitive function [17], we tested the major strains of *Lactobacillus* and *Bifidobacterium* [18] in this study.

Although there are numerous reports on probiotics’ beneficial effects, the underlying factors or mechanisms controlling probiotics’ effects on brain activity have not been specifically explored. Moreover, a few studies have reported their effects on the improvement of motor ability. To the best of our knowledge, no approach has been extended to analyze their effect combined with exercise intensity. Therefore, we hypothesized that the supplementation of probiotics, combined with exercise interventions, could facilitate motor function and brain activity. Thus, we aimed to assess the combined effect of the intake of probiotics and moderate- and high-intensity exercise on motor functions and levels of major inflammatory cytokines and neurotransmitters.

## 2. Materials and Methods

### 2.1. Animals

Four-week-old C57BL/6J male mice (*n* = 104) were acquired from Daehan Biolink (Eumsung, Korea) and divided into six groups: NX—no probiotics and no exercise, NM—no probiotics with moderate-intensity exercise, NH—no probiotics with high-intensity exercise, PX—probiotic ingestion without exercise, PM—probiotic ingestion with moderate-intensity exercise, and PH—probiotics ingestion with high-intensity exercise (*n* = 16–18 per group). The mice were kept in a room maintained at 20–24 °C with a constant 12 h light/12 h dark cycle with free access to water and food. All animal procedures were approved by the Institutional Animal Care and Use Committee (DKU18-018) and conformed to NIH guidelines.

### 2.2. Ingestion of Probiotics Solution

The probiotic solution was prepared by dissolving 2 g lyophilized powders of *L. plantarum* (1.2 × 10^9^ CFU) and *B. longum* (1.3 × 10^9^ CFU) (Human M tec., Cheonan, Korea) in 200 mL of water. On average, the amount of water consumed by a mouse per day was approximately 10 mL. Therefore, the final dose of *L. plantarum* and *B. longum* ingested was 1.2 × 10^8^ CFU and 1.3 × 10^8^ CFU per mouse per day, respectively. The average daily supplementation of probiotics per mouse from 4 to 8 weeks was then calculated.

### 2.3. Treadmill Exercise

Because the intensity and duration of exercise in the form of treadmill running could be controlled easily, unlike voluntary wheel running [19], here, we trained the mice using a treadmill (JD-A-09, Jung Do-B&P, Seoul, Korea). The intensity of exercise was set by speed control, as reported in earlier studies [20,21]. Briefly, the mice were trained at a speed of 2–3 m/min in the first week for moderate-intensity exercise, which was then increased to 10 m/min from the second to the fourth weeks. For high-intensity exercise, starting with 2 m/min, the speed was increased by 1 m/min every 2 min up to 18 m/min at 30 min. However, the intensity felt by each individual could vary; thus, as speed increased, the condition of the mice was more carefully monitored.

### 2.4. Rotarod Test

A rotarod test is widely used to assess rodents’ motor coordination, a well-known cerebellum-dependent behavior [22,23,24]. The test measures an animal’s ability to maintain balance on a rod that turns at an accelerating or a constant speed. Before starting a full-scale test, the mice were allowed to familiarize themselves with the experimenter and the experimental environment by handling. Then, they were trained for three days to learn the rotarod test. On the first day, the mice were made to run at 24 rpm for 60 s, which was increased to 28 rpm for 60 s on the second day, and 32 rpm for 60 s on the third day. The experiment was conducted at an accelerating and constant-speed mode for 1800 s using the rotarod (MED Associates Inc., Latham, New York, NY, USA). In an accelerating mode that measures the endurance, the mouse was placed on the stationary rod and then allowed to run by slowly increasing the speed of the rod from 4 rpm to 40 rpm. In the constant mode, the speed of the rotarod was set at 32 rpm for 1800 s, and the mice were placed onto the rotating rod. This mode measured motor coordination and needed more concentration. The rotarod tests were performed during the 31st–32nd (p31–32) and 59th–60th (p59–60) postnatal days. The tests were conducted twice with each mouse, and the average records of latency to fall were recorded. Expert skilled in animal behavior test over the years evaluated blinded experiment.

### 2.5. Tissue Preparation

On the 60th postnatal day (p60), at least 4 h after the end of the rotarod test, the mice were sacrificed. Subsequently, the mouse cerebellar tissue was extracted and rinsed with phosphate-buffered saline. It was then homogenized using tissue homogenizing agents (IKA, Königswinter, Germany), and then precipitated by adding equal volumes of lysis buffer in equal volume (as tissue). Subsequently, the solution was centrifuged at 13,000× *g* for 20 min at 4 °C, and the supernatants were transferred to a new tube. These were immediately sent for analysis or maintained at −70 °C before being analyzed.

### 2.6. Cytokines

Pro-inflammatory cytokine analysis was performed by a professional company (Komabiotech Inc., Seoul, Korea). The fluorescent intensity was measured after antigen-antibody reactions using a multi-flex kit capable of bead–antibody mixture reaction (Mouse magnetic Luminex assay kit, cat no. LXSAMSM-03, R&D Biosystems, Minneapolis, MS, USA). All assays were performed in duplicate.

### 2.7. GABA and Glutamate

The levels of GABA and glutamate were determined using liquid chromatography–mass spectrometry/mass spectrometry (LC-MS/MS) using an Agilent 1290 series rapid resolution LC system and a triple quadrupole linear ion trap mass spectrometer (4000 QTRAP) (AB Sciex, Foster City, CA, USA). The chromatographic separations were achieved using a Luna C8 column (100 mm × 2.0 mm, 3 μm, Phenomenex, Torrance, CA, USA). The mobile phases A and B consisted of 0.1% formic acid in water (LC-MS grade) and acetonitrile (LC-MS grade), respectively. The flow rate was 0.3 mL/min, and the injection volume was 10 μL. The mass spectrometer was optimized for the multiple reaction monitoring modes using electrospray ionization (ESI) in the positive mode. The ion spray voltage and the source temperature were set at 5000 V and 500 °C, respectively. The m/z transitions were set as 104.0 → 87.0 for GABA and 148.0 → 84.0 for glutamate. Data were acquired and analyzed using the software Analyst^®^ version 1.6 (AB Sciex, Foster City, CA, USA). Testing with known standards was performed to draw a calibration curve according to the standard internal method, and the concentrations of GABA and glutamate in the samples were obtained from the calibration curve.

### 2.8. Statistical Analysis

The Graphpad Prism ver. 8.4.3. Statistical Program was used for statistical analysis and data processing in this study. One-way analysis of variance (ANOVA) was used to analyze the effects of probiotic ingestion and treadmill exercise. The t-test was used to analyze the results of probiotic ingestion. A *p*-value of <0.05 was used to define the significant effects of treatments.

## 3. Results

### 3.1. Motor Function (Acceleration Mode and Constant-Speed Mode)

**Figure 1:**
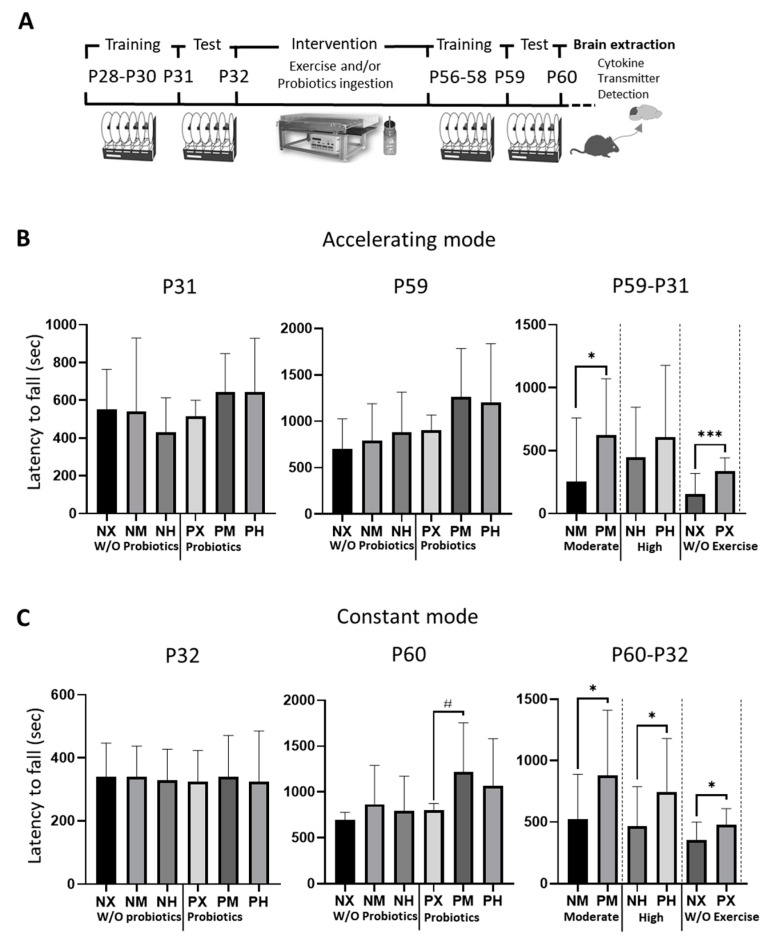
Effect of exercise and probiotics ingestion on motor function. Motor function is enhanced by probiotic ingestion. (**A**) Experimental timeline and schematic illustration. (**B**) Summary bar graphs showing latency to fall during test sessions of rotarod at accelerating mode. (**C**) Summary bar graphs showing latency to fall during test sessions of rotarod in constant mode. Values are presented as mean ± SD; #; *p* < 0.05. Significant difference was indicated by One-way ANOVA. *; *p* < 0.05, ***; *p* < 0.001. Significant difference was indicated by independent *t*-test. N, no probiotics; P, probiotics; M, moderate-intensity exercise; H, high-intensity exercise; X, no exercise.

In the acceleration mode, an uneven distribution was observed between different groups at p31 (Figure 1); however, there was no significant difference between groups. At p59, the PM group ran the longest (1264.9 s) (Table 1). When comparing the improvement of motor skills based on the presence of probiotics, the probiotic ingestion groups had longer latency to fall than those who did not take probiotics in the moderate-intensity and non-exercise groups (PX vs. NX: *p* = 0.038; PM vs. NM: *p* < 0.001). In the constant speed mode, which is fast from the beginning, unlike the acceleration mode, the mice did not adapt to the test easily because of a shortage of time. In the present study, records for the test at p32 showed an even distribution; however, at p60, mice in the PM group ran the longest (1219.7 s) (Table 2), showing significant differences from those in the PX group (*p* = 0.023) in terms of exercise intensity. The running times of the non-probiotic ingestion groups were in the order of NM > NH > NX groups. Moreover, the motor performance was improved in all the probiotic ingestion groups compared with those in the non-probiotic ingested groups (PM vs. NM: *p* = 0.036; PH vs. NH: *p* = 0.036, PX vs. NX: *p* = 0.012).

### 3.2. Cytokines

**Figure 2:**
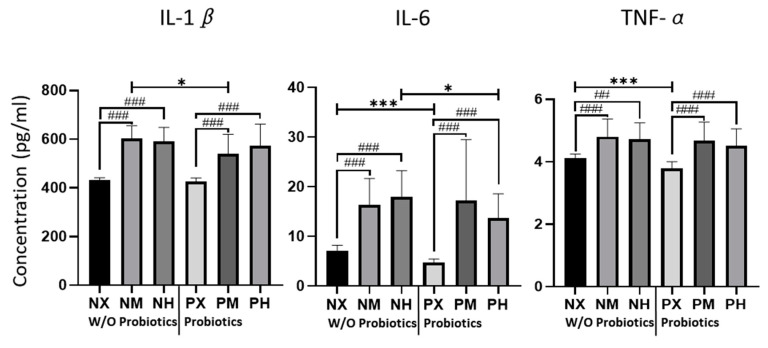
Effect of exercise and probiotics ingestion on inflammatory cytokines in the brain. Summary bar graphs showing the concentrations of IL-1β (left panel), IL-6 (middle panel), and TNF-α (right panel) in cerebellar lysate from mice brains. Cytokines were detected by ELISA (enzyme-linked immunosorbent assay). The cytokine level changes depending on the exercising intensity and probiotics intake of mice. Increasing or decreasing tend to be similar, regardless of the type of cytokine. Values are presented as mean ± SD, ##; *p* < 0.01, ###; *p* < 0.001. Significant difference was indicated by One-way ANOVA. *; *p* < 0.05, ***; *p* < 0.001. Significant difference was indicated by independent *t*-test. N, no probiotics; P, probiotics; M, moderate-intensity exercise; H, high-intensity exercise; X, no exercise.

The levels of inflammatory cytokines IL-1β, IL-6, and TNF-α, estimated from the mice brain tissue on p60, are shown in Figure 2. The NM and NH groups had the highest values of IL-1β (602.05 pg/mL, 590.43 pg/mL), which was lowest in the NX and PX groups (432.23 pg/mL, 426.14 pg/mL) (Table 3). Based on the exercise intensity, the NX group had significantly lower levels of IL-1β than NM and NH (*p* < 0.001), whereas it was significantly lower in the PX than in the PM and PH groups (*p* < 0.001). However, the effects of probiotics with moderate-intensity exercise lowered the levels of IL-1β (PM vs. NM: *p* = 0.015). The NH and PM groups showed the highest levels of IL-6 (17.92 pg/mL, 17.26 pg/mL), whereas the NX and PX groups showed the lowest values (7.13 pg/mL, 4.79 pg/mL). Based on the intensity of exercise, the IL-6 levels in NX were significantly lower than those in the NM and NH groups (*p* < 0.001). In addition, the IL-6 levels were significantly lower in the PX group than in the PM and PH groups (*p* < 0.001). Based on probiotic intake, the IL-6 levels of PX were lower than those of NX (*p* < 0.001), and the IL-6 levels of PH were lower than those of NH (*p* = 0.019). In terms of TNF-α, NM and NH showed the highest values (4.80 pg/mL, 4.71 pg/mL), and NX and PX had the lowest values (4.12 pg/mL, 3.78 pg/mL). Based on the intensity of exercise, the TNF- α level of NX was significantly lower than that of NM and NH (*p* < 0.001, *p* = 0.003). PX showed significantly lower values of TNF-α than PM and PH (*p* < 0.001), whereas probiotic intake significantly decreased the TNF-α content in PX compared to NX (*p* < 0.001).

### 3.3. GABA and Glutamate

**Figure 3:**
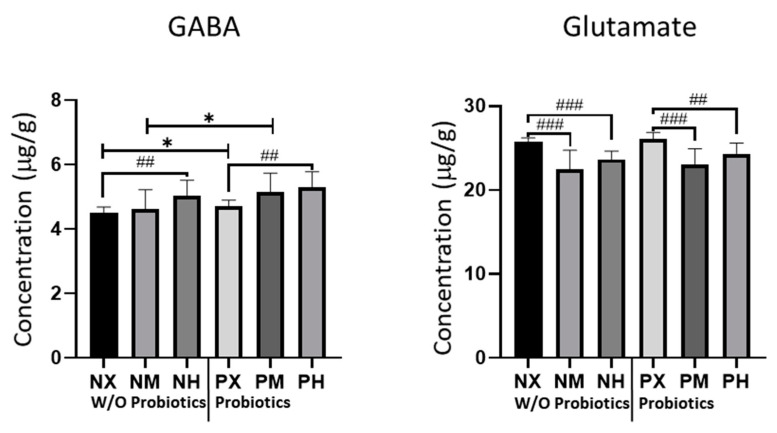
Effect of exercise and probiotics ingestion on major neurotransmitters of the brain. Summary bar graphs showing the concentrations of GABA (left panel) and glutamate (right panel) in cerebellar lysate from mice brains. GABA and glutamate were detected by LC-MS/MS. GABA concentrations are increased by ingestion of probiotics. GABA and glutamate concentrations are also affected slightly by exercise intensity. Values are presented as mean ± SD; ##; *p* < 0.01, ###; *p* < 0.001. The significant difference was indicated by One-way ANOVA. *; *p* < 0.05. Significant difference was indicated by independent *t*-test. N, no probiotics; P, probiotics; M, moderate-intensity exercise; H, high-intensity exercise; X, no exercise.

We measured the concentrations of GABA and glutamate in the cerebellum, which is the brain region responsible for motor coordination; their measured levels are shown in Figure 3. For GABA, the PH and PM groups had the highest values (5.30 μg/g, 5.15 μg/g), whereas the NM and NX groups had the lowest values (4.61 μg/g, 4.51 μg/g) (Table 4). Based on the intensity of exercise, NX and PX had significantly lower values of GABA than NH (*p* = 0.007) and PH (*p* = 0.002), respectively. Moreover, probiotic ingestion increased GABA levels in the PM and PX groups compared to the NM (*p* = 0.016) and NX (*p* = 0.003) groups. For glutamate, the PX and NX groups had the highest values (26.05 μg/g, 25.75 μg/g), whereas the NM and PM groups had the lowest values (22.45 μg/g, 23.09 μg/g). Based on the intensity of exercise, mice in the NX group had significantly higher glutamate levels than those in the NM and NH groups (*p* < 0.001), while the PX group showed significantly higher levels of glutamate than the PM and PH groups (*p* < 0.001 and *p* = 0.005, respectively). These results indicated that probiotic intake did not significantly affect the concentrations of glutamate.

## 4. Discussion

In this study, we sought to investigate the effects of aerobic exercise and probiotic intake from childhood to adulthood on motor skills and levels of cytokines and neurotransmitters in the brain. In terms of motor ability, the rotarod test performed at p31 to p32 did not show any significant differences, which could be ascribed to the lower adaptability of the young mice right after separation from their mother to the new environment, and their unfamiliarity with the testing. However, after about four weeks of probiotic ingestion with moderate- or high-intensity aerobic exercise from p31 to p60, the trial on p60 showed a distinct difference between the groups. Notably, it showed significant results not only in the acceleration mode but also in the constant-speed mode. Taken together, these results confirmed the positive effects of probiotics on both endurance and motor coordination. Previous studies have shown that probiotics might contribute to the nervous system’s balance by increasing the production of GABA [25], and in the recovery of post-exercise inflammation responses, such as cytokine regulation [26]. Moreover, probiotic intake has been reported to relieve symptoms of depression or anxiety [27,28], and in the present study, the probiotic intake group showed better motor skills than the control group. This could be ascribed to the positive cognitive effect of probiotic intake that helped the mice overcome anxiety or fear during exercise.

A few studies have reported the beneficial effects of exercise and probiotics on inflammatory cytokine response. For instance, Huang et al. showed that TNF-α and IL-6 in athletes decreased after *Lactobacillus* intake for 14 weeks [8]. Moreover, some studies have shown that human patients suffering from chronic inflammation responded positively to the ingestion of probiotics, as the latter decreased the production of TNF-α [29,30].

In the present study, we observed a significant increase in GABA levels in the brain with increasing exercise intensity, whereby the high-intensity exercise groups recorded the highest concentrations. These results are congruent with an earlier report that showed a 20% increase in GABA in the brain’s sensory–motor cortex after high-intensity exercise [31]. In addition, studies reporting the changes in neurotransmitters in various areas of the cerebral cortex after high-intensity exercise in which a person reaches more than 80% of the maximum heart rate showed that the amount of GABA is increased across the cerebral cortex, including the motor cortex responsible for the motor function, the area responsible for the execution function, the visual cortex, and the cingulate cortex [32].

Furthermore, the present study revealed that an improvement in the probiotic ingestion group’s motor function with moderate-intensity exercise was more significant than that of the group trained for high-intensity exercise, which could be assumed to be an adverse effect of high-intensity exercise. Earlier studies have reported similar adverse effects, such as the increased risk of infection [3], chronic fatigue, and reduced motor performance [5]. Besides this, improper exercise intensity or strenuous exercise also increases oxidative stress and active oxygen [33]. However, from the present study results, there is a likelihood that probiotic supplementation could decrease the level of inflammatory cytokines more than the level of non-inflammatory cytokines, consequently alleviating the side effects of high-intensity exercises, such as inflammatory reactions.

One of the limitations of this study is the lack of information on whether probiotics treatment altered the gut microbiota. In addition, there is no direct evidence for the causal relationship that asserts that the effects of probiotic intake and aerobic exercise are responsible for the improvement in motor function and the increase in cytokine levels observed in this study. Therefore, further studies are required to investigate the significant clues of gut microbiota changes after probiotics ingestion, and the molecular mechanisms.

## 5. Conclusions

Exercise during adolescence has many positive effects; however, it is associated with acute inflammation risks, which necessitate the search for a proper way to exercise. We hypothesized that probiotic ingestion could solve the adverse effects of exercise, and investigated the effect of exercise and probiotic ingestion on the brain’s immunity and neurotransmitters. The study showed the most considerable motor function improvement and the lowest cytokine level in the mouse group subjected to both exercise and probiotic ingestion, suggesting that exercise from childhood to adolescence is necessary. Taking supplements such as probiotics could avoid inflammatory reactions and improve motor functions. These findings could help in developing nutritional strategies to enhance exercise adaptations, leading to improved health and youth performance.

## Figures and Tables

**Table 1:** Statistical values of latency to fall from the rotarod test at acceleration mode. Values are presented as M (mean, sec) and SD (Standard Deviation). At P31 and P59, significant difference was assessed by One-way ANOVA at P31 and P59. For the improvement assessment, the significant difference was evaluated by independent *t*-test at P59-P31. N, no probiotics; P, probiotics; M, moderate-intensity exercise; H, high-intensity exercise; X, no exercise.

Acceleration Mode (Endurance)	GROUP	*n*	M (sec)	SD	F	T-test F
P31 (Pre)	NM	16	542.31	387.85	1.979	
	PM	16	643.63	204.49		
	NH	18	430.44	182.13		
	PH	18	643.83	284.76		
	NX	18	551.33	212.01		
	PX	18	516.33	83.808		
P59 (Post)	NM	16	796.25	393.74	4.530	
	PM	16	1264.9	518.53		
	NH	18	879.00	434.58		
	PH	18	1201.9	634.57		
	NX	18	706.00	321.69		
	PX	18	900.22	167.54		
P59-P31 (Improvement)	NM	16	253.94	505.46		1.255
	PM	16	621.25	451.19		
	NH	18	448.56	397.24		2.074
	PH	18	606.06	572.07		
	NX	18	154.67	163.36		2.566
	PX	18	339.44	101.97		

**Table 2:** Statistical values of latency to fall from the rotarod test at constant mode. Values are presented as M (mean, sec) and SD (Standard Deviation). At P32 and P60, significant difference was assessed by One-way ANOVA at P31 and P59. For the improvement assessment, the significant difference was evaluated by independent *t*-test at P60-P32. N, no probiotics; P, probiotics; M, moderate-intensity exercise; H, high-intensity exercise; X, no exercise.

Constant Mode (Motor Coordination)	GROUP	*n*	M (sec)	SD	F	T-test F
P32 (Pre)	NM	16	340.69	96.69	0.082	
	PM	16	340.44	130.38		
	NH	18	329.00	98.37		
	PH	18	323.89	161.21		
	NX	18	340.44	106.53		
	PX	18	325.22	97.86		
P60 (Post)	NM	16	866.25	425.38	4.562	
	PM	16	1219.7	535.25		
	NH	18	794.89	376.80		
	PH	18	1069.3	512.76		
	NX	18	696.67	80.63		
	PX	18	803.33	70.98		
P60-P32 (Improvement)	NM	16	525.56	364.36		2.139
	PM	16	879.25	532.89		
	NH	18	465.89	322.65		1.818
	PH	18	745.44	435.02		
	NX	18	356.22	143.04		1.190
	PX	18	478.11	131.12		

**Table 3:** Statistical values of cytokines for the concentrations of IL-1β (left panel), IL-6 (middle panel), and TNF-α (right panel) in cerebellar lysate from mice brains. Cytokines were detected by ELISA. Values are presented as M (mean, pg/mL) and SD (Standard Deviation). N, no probiotics; P, probiotics; M, moderate-intensity exercise; H, high-intensity exercise; X, no exercise.

Analytes	GROUP	*n*	M (ρg/mL)	SD	F	T-test F
IL-1β	NM	16	602.05	53.00	32.38	2.214
	PM	16	540.53	78.85		
	NH	18	590.43	58.16		2.326
	PH	18	573.08	88.69		
	NX	18	432.23	9.25		2.658
	PX	18	426.14	15.09		
IL-6	NM	16	16.34	5.31	15.02	5.302
	PM	16	17.26	12.23		
	NH	18	17.92	5.34		2.215
	PH	18	13.72	4.84		
	NX	18	7.13	1.07		2.748
	PX	18	4.79	0.64		
TNF-α	NM	16	4.80	0.57	13.15	1.069
	PM	16	4.68	0.58		
	NH	18	4.71	0.54		1.030
	PH	18	4.51	0.54		
	NX	18	4.12	0.13		2.802
	PX	18	3.78	0.22		

**Table 4:** Statistical values of major neurotransmitters in brain. GABA and glutamate were detected by LC-MS/MS. Values are presented as M (mean, μg/g) and SD (Standard Deviation). One-way ANOVA evaluated the significant difference. N, no probiotics; P, probiotics; M, moderate-intensity exercise; H, high-intensity exercise; X, no exercise.

Analytes	GROUP	n	M (μg/g)	SD	F	T-test F
GABA	NM	16	4.61	0.60	8.87	1.065
	PM	16	5.15	0.59		
	NH	18	5.05	0.47		1.070
	PH	18	5.30	0.49		
	NX	18	4.51	0.17		1.198
	PX	18	4.71	0.19		
Glutamate	NM	16	22.45	2.29	18.84	1.556
	PM	16	23.09	1.83		
	NH	18	23.66	0.97		1.648
	PH	18	24.35	1.24		
	NX	18	25.75	0.47		3.033
	PX	18	26.05	0.81		

## Data Availability

The datasets used during the current study are available from the corresponding author upon reasonable request.
